# A Markov chain-based model for structural vulnerability assessmentof corrosion-damaged reinforced concrete bridges

**DOI:** 10.1098/rsta.2020.0290

**Published:** 2021-08-09

**Authors:** Ebrahim Afsar Dizaj, Jamie E. Padgett, Mohammad M. Kashani

**Affiliations:** ^1^ Department of Civil Engineering, Azarbaijan Shahid Madani University, Tabriz, Iran; ^2^ Department of Civil and Environmental Engineering, Rice University, Houston, TX 77005, USA; ^3^ Faculty of Engineering and Physical Sciences, University of Southampton, Southampton SO17 1BJ, UK

**Keywords:** Markov chain, corrosion, bridge, reinforced concrete, concrete crack, vulnerability

## Abstract

The deterioration and cracking of reinforced concrete (RC) bridges due to the chloride-induced corrosion of steel reinforcement is an inherently time-dependent stochastic phenomenon. In the current practice of bridge management systems, however, the determination of the condition states of deteriorated bridges is highly dependent on the opinion of experienced inspectors. Taking such complexity into account, the current paper presents a new stochastic predictive methodology using a non-homogeneous Markov process, which directly relates the visual inspection data (corrosion rate and crack widths) to the structural vulnerability of deteriorated concrete bridges. This methodology predicts the future condition of corrosion-induced damage (concrete cracking) by linking structural vulnerability analysis and a discrete-time Markov chain model. The application of the proposed methodology is demonstrated through a case-study corrosion-damaged RC bridge pier.

This article is part of a discussion meeting issue ‘A cracking approach to inventing new tough materials: fracture stranger than friction’.

## 1. Introduction

Chloride-induced corrosion of reinforcing steel, due to aggressive coastal areas or de-icing salt in winter, is the most significant environmental threat affecting the performance of ageing reinforced concrete (RC) structures and bridges worldwide [1]. An investigation on just 1/10 of the total UK bridge inventory revealed that the annual cost of corrosion damage to RC bridges located in England and Wales is estimated to be about 1 billion pounds [2]. In the USA, the total estimated direct cost to repair ageing infrastructure is over 200 billion dollars [3].

Once chloride-induced corrosion initiates in reinforcing bars, the cumulative expansion of corrosion products results in degradation of concrete as well as non-uniform pitting of reinforcing bars in RC structures. At the initial stage of corrosion, the non-uniform pitting corrosion is hidden until the accumulation of corrosion products results in concrete cover cracking. Therefore, there is large uncertainty in predicting the condition state (CS) of ageing RC structures. As such, evaluating the structural performance of RC structures subjected to continuous (time-dependent) chloride-induced deterioration requires explicit consideration of the uncertainty in the state of the structure, proactive rehabilitation and maintenance decision-making. The corrosion of reinforcing bars, however, is an underlying continuous process in its initial stages, and cannot be observed directly. To evaluate the condition of an existing RC structure that is located in an aggressive environment, knowledge on the amount of corrosion level (mass loss of reinforcement) is necessary. After corrosion initiation, the mass loss of reinforcement can be estimated using the corrosion rate measurement test [4,5]. Subsequently, using available models, time to crack initiation [6,7] and crack propagation [8,9] can be predicted. If the structure has passed this stage and has already cracked, the crack width and corrosion rate can be measured on site and the growth of crack width can be estimated using available models [10–12].

There are several models available in the literature to predict the time to corrosion-induced concrete cover crack initiation. These models can be divided into numerical models (fracture mechanics-based) [6,13–15], empirical models [7,16,17] and analytical models [18–21]. Moreover, there are several models in the literature to estimate the relationship between the width of corrosion-induced cracks and cross-sectional area loss (or mass loss) of reinforcement [10–12,22–25].

Discrete-time Markov chain models have been leveraged for sequential engineering decision problems, where the decision should be determined at discrete time intervals [26]. In this approach, the predicted future state of a system depends only on its present state, i.e. is independent of any past conditions [27]. Markov chain-based solutions have been recently used in several civil engineering problems [28–33], in particular within the optimization of maintenance and rehabilitation of highway transport infrastructure [34,35]. Owing to its strategic advantages, Markovian discrete-state deterioration models have been widely used in management systems of highway bridges in the USA [36,37].

In the current practice of bridge management system (BMS), Markov chain models are employed to determine the probability of transitioning between consecutive CSs to assess the rate or degree of deterioration, where the CSs are assigned to the bridge elements by the subjective assessment of suitably qualified inspectors [38]. The current Markovian deterioration models, however, are only based on visual inspection data without any direct relationship to the structural performance [38]. Probabilistic structural vulnerability analysis (SVA) [39,40] is an effective method that accounts for uncertainties associated with structural materials, geometry, environmental condition and loading. Combining a detailed SVA with a Markov chain-based predictive model creates a link between visual inspection data and residual structural performance of ageing RC structures and bridges.

### (a) Research novelty and contribution

The above discussion shows that currently, the determination of the future condition of deteriorated structures is highly dependent on the opinion of the experienced inspectors. Therefore, there is a significant paucity in the literature to directly quantify and predict the relationship between the observable extent of deterioration (i.e. corrosion rate and corrosion-induced concrete cracking) and the structural performance of ageing bridges through a generic stochastic predictive model. Using a Markov chain-based deterioration model, this paper aims to create a stochastic platform to predict the time-variant performance limit states using SVA of corroded RC bridges, which links the visual inspection data to the numerically simulated residual structural capacity.

**Figure 1.**
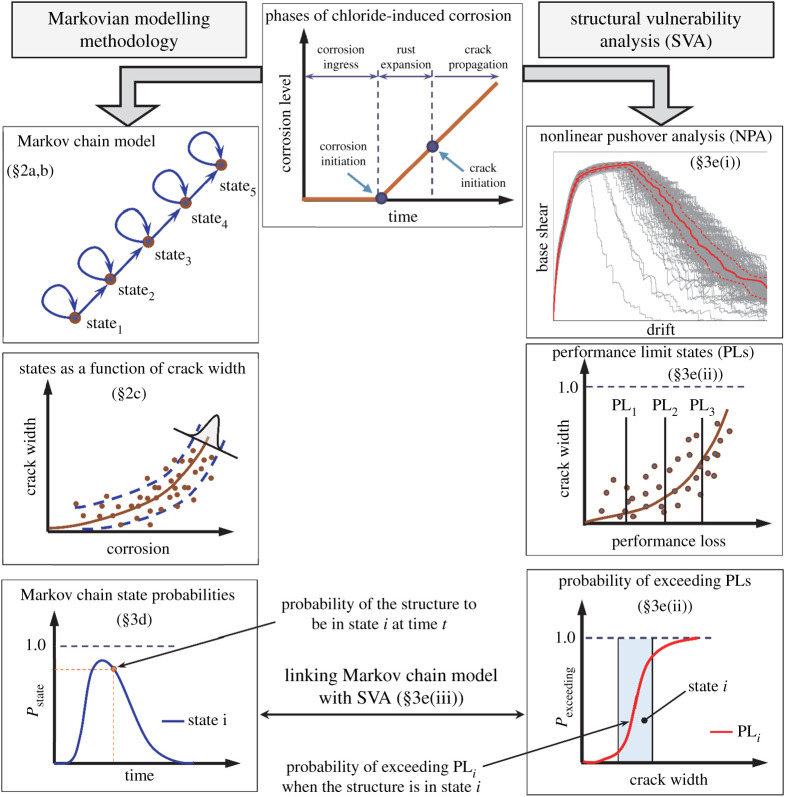
Overview of the proposed framework and interrelationship between the discrete-time Markov chain model and SVA. (Online version in colour.)

Figure 1 shows an overview of the proposed methodology and interrelationship between Markov chain sates and SVA. The proposed methodology employs a state-based discrete-time Markov chain model, where the CSs of the system are defined based on a possible range of visible corrosion-induced crack sizes. This model is described in detail in §2a,b. In §2c, the empirical relationships to estimate the time since corrosion initiation, as well as corrosion degree through the observable corrosion-induced crack width, are derived. Subsequently, the application of the proposed methodology is demonstrated in §3 through a case-study structure. First, the structural details and the finite-element model of the case-study structure are presented in §3a,b; then, the random variables (deterioration model, structural details) are simulated using Monte Carlo simulation (§3c). In §3d, the probability of being in each CSs (defined in §2b) is estimated as a function of time from corrosion initiation. Subsequently, in §3e(i) and (ii), the residual structural capacity (in terms of ductility and strength) is evaluated for a wide range of increasing corrosion-induced crack sizes. Finally, in §3e(iii), a conceptual relationship is established between the state probabilities estimated based on the proposed Markov chain model and predefined structural performance limit states through SVA.

It is important to note that the main focus of the current study is on the chloride-induced corrosion of reinforcements, where other sources of corrosion such as carbonation-induced corrosion and corrosion due to using calcium chloride as an admixture in concrete mix are not relevant.

## 2. Probabilistic modelling methodology

### (a) Description of the proposed Markov chain model

The degradation of concrete bridges is an inherently time-dependent stochastic phenomenon. Taking such complexity into account, in this paper, the CS of corroded bridges is defined as a discrete-time stochastic process. More specifically, the prediction of the future CS is abstracted as a discrete-time Markov chain. The general concept of a discrete-time Markov chain model is presented in the below equation

2.1 $$P(X_{n+1}=j|X_{n}=i,X_{n-1}=i_{n-1},\ldots ,X_{0}=i_{0})=P(X_{n+1}=j|X_{n}=i)=P_{ij},$$

where *X*_1_, *X*_2_, …. , *X_n_* are random variables with the Markovian property, i.e. the probability of moving to the future CS *j* depends only on the present CS *i* and is independent of any previous CSs (*i_n_*_−1_, *i_n_*_−2_, … , *i*_0_). *P_ij_* is the one-step transition probability and represents the probability that the chain, given that the current CS is *i*, moves to the next into CS *j* (an increment of time later). Thus, in this study, the future CS of the bridge is estimated given thepresent CS.

In a Markov chain approach, the transition probability of a given bridge from one CS to another is required [41]. Since corrosion is a time-dependent process, in the current study, the probability of transitioning between the *N* predefined CSs is considered through a time-variant Markov transition probability matrix as defined in the below equation

2.2 $$P(t,t+\Delta )=\left[\begin{array}{cccc} P_{11}(t,t+\Delta ) & P_{12}(t,t+\Delta ) & \ldots . & P_{1N}(t,t+\Delta )\\ 0 & P_{22}(t,t+\Delta ) & \ldots . & P_{2N}(t,t+\Delta )\\ . & . & \ldots . & .\\ . & . & \ldots . & .\\ . & . & \ldots . & .\\ 0 & 0 & \ldots . & P_{NN}(t,t+\Delta ) \end{array}\right].$$

where *P_ij_* (*t*, *t* + Δ) is the transition probability of the system from predefined CS *i* at time *t* (in this study the time *t* =* t_p_*, where *t_p_* is the time from corrosion initiation) to the CS *j* (at time *t* + Δ) and Δ is the considered time increment. In other words, we evaluate the CS via a non-homogeneous Markov process. The transition probability matrix defined in equation (2.2) is upper triangular because it is assumed that during the time window (*t*, *t* + Δ), no repair or rehabilitation will take place to restore the bridge to a less severe condition. Assuming that the structure can either remain in its current CS or just move to the next CS during a given time window (considering small time increment), the Markov transition probability matrix presented in equation (2.2) will become a bivariate case as defined in the below equation

2.3 $$P(t,t+\Delta )=\left[\begin{array}{ccccc} P_{11}(t,t+\Delta ) & 1-P_{11}(t,t+\Delta ) & 0 & \ldots & 0\\ 0 & P_{22}(t,t+\Delta ) & 1-P_{22}(t,t+\Delta ) & \ldots & 0\\ . & . & . & \ldots & 0\\ . & . & . & \ldots & 0\\ . & . & . & \ldots & 0\\ 0 & 0 & . & \ldots & 1 \end{array}\right].$$

To estimate the elements of the transition probability matrix, several approaches have been posed in the literature, such as adopting a Poisson regression model [42], ordered probit model [43] or deriving them based on stochastic duration model [44].

In this study, a similar approach to the duration model proposed by Mishalani & Madanat [44] is used to construct the Markov chain transition probability matrix (equation (2.3)) at each time increment. Based on this approach, the conditional probability of transitioning out of a specific CS is calculated as

2.4 $$R_{i}(t,\Delta )=P(t<T_{i}<t+\Delta |T_{i}>t)=\frac{F_{i}(t+\Delta )-F_{i}(t)}{1-F_{i}(t)}.$$

where *R_i_*(*t*, Δ) is the probability of transitioning out of CS *i* at the time window of (*t*, *t *+ Δ), given that at time *t*, the system is in the CS *i*, and *T_i_* is the duration of the CS *i* which is a random variable. *F_i_*(t) is the cumulative distribution function of *T_i_* which is simulated based on the threshold of leaving CS *i*. In §3d, a procedure for characterizing the *F_i_*(t) is presented. Therefore, with the cumulative probability distribution function of *T_i_*, the elements of the Markov transition probability matrix (equation (2.3)) of the corresponding discrete-time period can be calculated as follows:

2.5 $$P(t,t+\Delta )=\left[\begin{array}{ccccc} 1-R_{1}(t,\Delta ) & R_{1}(t,\Delta ) & 0 & \ldots & 0\\ 0 & 1-R_{2}(t,\Delta ) & R_{2}(t,\Delta ) & \ldots & 0\\ . & . & . & \ldots & 0\\ . & . & . & \ldots & 0\\ . & . & . & \ldots & 0\\ 0 & 0 & . & \ldots & 1 \end{array}\right].$$

Once the transition probabilities are calculated for each time interval, the probability of the system being in each predefined CS (i.e. CS probability) can be calculated by the product in the below equation

2.6 $$\left[\begin{array}{cccc} P_{s}^{1}(t+\Delta ) & P_{s}^{2}(t+\Delta ) & \ldots & P_{s}^{N}(t+\Delta ) \end{array}\right]=\left[\begin{array}{cccc} P_{s}^{1}(t) & P_{s}^{2}(t) & \ldots & P_{s}^{N}(t) \end{array}\right]\times P(t,t+\Delta ),$$

where $P_{s}^{1}(t+\Delta )$, ${P_{s}}^{2}(t+\Delta )$ and ${P_{s}}^{N}(t+\Delta )$ are the probability of a system being in CS_1_, CS_2_ and CS *N*, respectively, at time *t* + Δ and ${P_{s}}^{1}(t)$, ${P_{s}}^{2}(t)$ and ${P_{s}}^{N}(t)$ are the corresponding CS probabilities at time *t*. For the initial condition (i.e. for *t *= *t_p_* = 0), where the system is sound, the transition probabilities can be represented by a 1 × *N* row matrix as follows:

2.7 $$\left[\begin{array}{cccc} P_{s}^{1}(t=0) & P_{s}^{2}(t=0) & \ldots & P_{s}^{N}(t=0) \end{array}\right]=\left[\begin{array}{cccc} 1 & 0 & \ldots & 0 \end{array}\right].$$

The above equation indicates that at *t *= 0, the system is in CS_1_. However, depending on the current condition, the system might be in a different CS. For example, if inspection data show that the system is already corroded, depending on the level of corrosion, the current CS can be estimated. In this study, CSs are considered based on the corrosion-induced crack width (*w*). The proposed CSs are defined in the following section.

### (b) Defining the crack-based condition states

With a Markov chain model adapted to represent the CS of bridges, the system CSs are then defined in the context of corrosion-induced cracking of corroded RC components. Various crack width-based criteria have been suggested for condition limit states of RC structures in the literature. Washington State Bridge Inspection Manual [45], as well as Vu *et al*. [23], suggest a crack width of 1–3 mm as the service failure limit state of RC members. Val [46] suggested a crack width of 0.5 mm as the serviceability limit state for corroded RC structures. A wide range of references [17,47–49] suggest a crack width of 0.3–0.4 mm as the durability limit state and a crack width of 0.8–1 mm as the serviceability limit state. AASHTO guide specifications for LRFD seismic bridge design [50] recommend the crack width of up to 1.6 mm as the good condition (CS_1_) for the structural components of concrete bridges (slabs, decks, beams and piers). Moreover, this specification suggests a crack width of 1.6–3.2 mm as the fair condition (CS_2_), and crack width greater than 3.2 mm as the poor condition (CS_3_).

The above discussion reveals that consensus is lacking on linking a specific crack width to a specific CS. Therefore, the current study employs representative CSs mapped to a wide range of corrosion-induced crack widths; and then, the probability of being in each CS is related to structural capacity (in terms of the ductility and strength) as described in §3e. Table 1 shows definitions of the proposed CSs, where CS_1_ covers the period from the onset of corrosion to the onset of cracking (i.e. 0 < *t_p _*≤ *t*_cr_, where *t*_cr_ is the crack initiation time), i.e. the period that *w *= 0 when the structure remains in an uncracked condition. The CS_2_ is the condition that corrosion-induced crack width is between 0 and 0.5 mm. The CS_3_, CS_4_ and CS_5_ are similarly defined based on the crack width as indicated in table 1. If *w* > 3 mm, system is severely damaged due to concrete cover spalling and excessive corrosion, and therefore, it requires immediate action. This assumption is verified by experimental tests in a recent review paper [51] as well as §3e(i) by conducting nonlinear pushover analysis (NPA) and SVA on a case-study corroded RC bridge pier.

**Table 1.** CS definition.

condition state	symbol	condition state description
condition state 1	CS_1_	*w *= 0
condition state 2	CS_2_	0 <* w* ≤ 0.5 mm
condition state 3	CS_3_	0.5 <* w* ≤ 1 mm
condition state 4	CS_4_	1 <* w* ≤ 2 mm
condition state 5	CS_5_	2 <* w* ≤ 3 mm

The CS thresholds defined in table 1 will be used in §3d to estimate the CS probabilities. In the next section, based on an existing empirical model, a relationship is derived to estimate the degree of corrosion (in terms of mass loss percentage) from the observed corrosion-induced crack width.

### (c) Corrosion-induced concrete cover cracking

**Figure 2.**
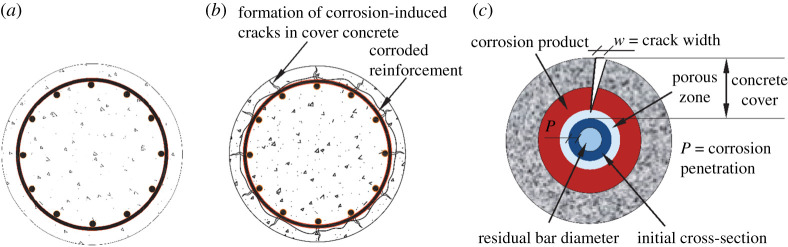
Schematic stages of corrosion-induced cover concrete cracking: (*a*) uncracked section; (*b*) development of microcracks; and (*c*) excessive cracking and interface around a corroded rebar with concrete. (Online version in colour.)

After a period from corrosion initiation, the accumulation of expansive corrosion products around the reinforcement results in corrosion-induced cracks alongside the longitudinal reinforcement (figure 2). The propagation of these cracks results in expediting the rate of chloride and moisture ingress, and finally spalling of the cover concrete. Figure 2 shows the process of formation and propagation of corrosion-induced cover cracks in a circular RC bridge pier section.

Among the existing models in the literature, the approach proposed by Liu & Weyers [18] has been widely used and verified by other researchers [46,52]. Using this model, the elapsed time from corrosion initiation to corrosion-induced crack formation *t*_cr_ can be estimated as

2.8 $$t_{\text{cr}}=\frac{W_{\text{cr}}^{2}}{2k_{p}},$$

where *W*_cr_ is the critical amount of corrosion products to cause concrete cover cracking and *k_p_* is the rate of rust production which can be obtained from the following equation [53]

2.9 $$k_{p}=0.00383d_{l}i_{\text{corr},l},$$

where *d_l_* is the diameter of longitudinal reinforcement and *i*_corr*,l*_ is the corrosion current density of longitudinal reinforcement at the onset of corrosion initiation, which can be obtained employing the empirical equation proposed in [54]. Further details on the calculation of *t*_cr_ are available in [46].

Vidal *et al*. [8] investigated two naturally corroded beam specimens (over 14 and 17 years) in a saline environment and found that there is a linear relationship between the amount of corrosion and the corrosion-induced crack width (*w*). This model has been widely adopted by other researchers in durability and life cycle analysis of deteriorated RC structures [46,55,56]. Therefore, in this study, this model is employed to relate the width of corrosion-induced crack to the mass loss percentage of longitudinal bars

2.10 $$w=0.0575\;(\Delta A_{s}-\Delta A_{s0}),$$

where Δ*A_s_* is the cross-sectional loss of reinforcement (in mm^2^) and Δ*A_s0_* is the cross-sectional loss of reinforcement needed for crack initiation (in mm^2^). Δ*A_s_*−Δ*A_s0_* can be obtained by the following equation [46]:

2.11 $$\Delta A_{s}-\Delta A_{s0}=\pi \Delta P(t)[d_{l}-2P_{0}-\Delta P(t)],$$

where Δ*P*(*t*) is the additional penetration of corrosion at time *t* after crack initiation (equation (2.12)) and *P*_0_ is the penetration of corrosion corresponding to Δ*A_s0_* (equation (2.13))

2.12 $$\Delta P(t)=0.0116i_{\text{corr},l}(t_{p}-t_{\text{cr}}),$$

and

2.13 $$P_{0}=\frac{\alpha W_{\text{cr}}}{\pi d_{l}\rho_{\text{steel}}},$$

where *t_p_* is the time from corrosion initiation (in years); *α* is the ratio between the molecular weight of steel and molecular weight of corrosion products, which can be taken as 0.57 [46], and *ρ*_steel_ is the density of steel. Substituting equations (2.11) to (2.13) to equation (2.10), the crack width can be calculated using the below equation

2.14 $$w=0.002i_{\text{corr},l}(t_{p}-t_{\text{cr}})\times [d_{l}-2P_{0}-0.0116i_{\text{corr},l}(t_{p}-t_{\text{cr}})].$$

From equation (2.14), *t_p_* can be obtained as a function of *w*

2.15 $$t_{p}(w)=\frac{F-\sqrt{\Delta }}{2E}+t_{\text{cr}},$$

where *F* = 0.002*i*_corr*,l*_ (*d_l_ − 2P_0_*); *E* = 2.32 × 10^−5^*i*^2^_corr*,l*_ and Δ = *F*^2^–4*Ew*.

Equation (2.15) will be used in §3d to simulate the *F_i_*(*t*) (equation (2.4)) which is needed to estimate the CS probabilities. Having *t_p_*(*w*), the corresponding average corrosion penetration (*P*_ave_(*w*)) can be obtained through equation (2.16):

2.16 $$P_{\text{ave}}(w)=\kappa \int_{0}^{t_{p}}i_{\text{corr}}(t_{p})\;\text{d}t_{p},$$

where *κ* is a coefficient equals to 0.0116, and *i*_corr_(*t_p_*) is the time-dependent corrosion rate according to the empirical equation proposed by Vu & Stewart [54]

2.17 $$i_{\text{corr}}(t_{p})=0.85i_{\text{corr},l}{t_{p}}^{-0.29}.$$

Finally, having *P*_ave_(*w*) for each crack size, the crack-width-dependent mass loss percentage of reinforcing bars (*ψ*(*w*)) can be calculated from the below equation

2.18 $$\psi (w)=\left[1-{\left(\frac{d_{l}-2P_{\text{ave}}(w)}{d_{l}}\right)}^{2}\right]\times 100.$$

The next section describes the approach proposed herein to relate the predefined corrosion-induced crack width-based CSs (presented in table 1) to the structural performance limit states of a given corrosion-damaged RC structure.

### (d) The relationship between predefined condition states and structural performance

**Figure 3.**
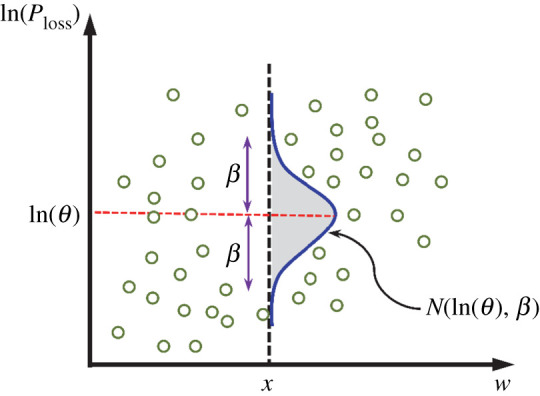
Schematic quantification of structural performance loss versus crack width. (Online version in colour.)

An SVA methodology is proposed here to find a relationship between the CS probabilities (the probability of being in predefined CSs in table 1) and the structural performance of corroded structures. This methodology has been used in the earthquake engineering, but in a different context [57,58]. According to the proposed methodology, the response of the corroded structure is analysed at different corrosion levels (corresponding to a specific range of *w*) using the NPA. Using NPA results, the performance loss of the structure (due to the negative impact of corrosion) is quantified in terms of ductility loss (*μ*_loss_) and strength loss (*S*_loss_). Figure 3 shows the structural performance loss (*P*_loss_) versus the progressive size of crack width schematically. Moreover, a distribution fit (here the lognormal distribution) of the performance loss values corresponding to a given corrosion-induced crack width (*w *= *x*) is mapped to this figure. The definition of PLs is presented in §3e(i) for the case-study structure.

Using the results of the NPA (in the schematic form shown in figure 3), the probability of exceeding a specific performance limit state can be obtained from the below equation

2.19 $$P[P_{\text{loss}}\geq \text{P}{\text{L}}_{i}|w=x]=1-\Phi \left(\frac{\ln(\text{P}{\text{L}}_{i})-\ln(\theta )}{\beta }\right),$$

where *P*_loss_ is the performance loss (either *μ*_loss_ or *S*_loss_) and *P*[.] is the probability that *P*_loss_ exceeds the leaving threshold of the *i*th performance limit state (PL*_i_*), given that the width of corrosion-induced crack (*w*) equals *x*. The form shown in equation (2.19) suggests that for a given *w*, the estimated values of *P*_loss_ follow a lognormal distribution where *Φ*(.) is the normal cumulative distribution function, ln(*θ*) is the logarithmic mean (equation (2.20)) and *β* is the logarithmic standard deviation (equation (2.21)) of the estimated values of *P*_loss_

2.20 $$\ln(\theta )=\frac{\sum_{i=1}^{n}\ln({P_{\text{loss,}}}_{i})}{n}$$

and

2.21 $$\beta =\sqrt{\frac{\sum_{i=1}^{n}{\left(\ln({P_{\text{loss,}}}_{i})-\ln(\theta )\right)}^{2}}{n-1}}.$$

In the above equations, *n* is the number of simulations (number of random samples) and *P*_loss*,i*_ is the value of the performance loss (induced by corrosion) for the *i*th combination of the random variables corresponding to *w = x*. It should be noted that the hypothetical random variables of the current study are defined in §3c for the considered case-study structure. The adequacy of the lognormal distribution fit will be demonstrated in §3e(ii). Using equation (2.19), the probability of exceeding each predefined PL can be estimated for crack width thresholds of predefined CSs (as defined in table 1), and, therefore, it can be related to the CS probabilities. In §3e(ii), this conceptual relationship is quantitatively presented for an exemplary case-study structure.

In the next section, the application of the proposed methodology is investigated on a corroded RC bridge pier.

## 3. Case study on a corroded RC bridge pier

**Figure 4.**
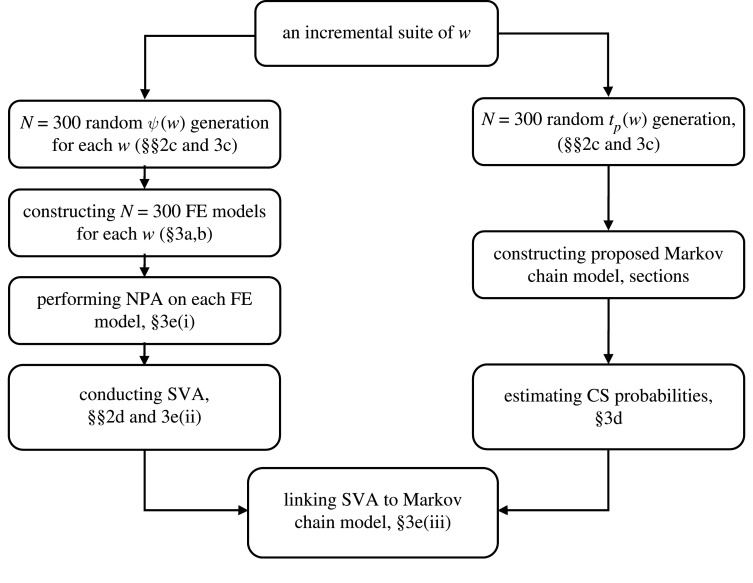
Considered procedure for the implementation of the proposed methodology on a case-study structure.

The methodology presented in §2 is demonstrated through a case-study structure in this section. Figure 4 shows the phases of the proposed procedure. As it is shown in figure 4, the procedure is divided into two parallel flows: (i) SVA as defined in §2d (left side of the flowchart), and (ii) Markov chain-based prediction model for corrosion-induced cracked structure as defined in §2a (right side of the flowchart). These two parallel procedures are employed to develop a relationship between SVA (as defined in §2d) and the CS probabilities, CS (as defined in §2a,b), which can be used in decision-making processes related to bridge management and maintenance, among other potential applications.

### (a) Proposed case-study RC bridge pier

**Figure 5.**
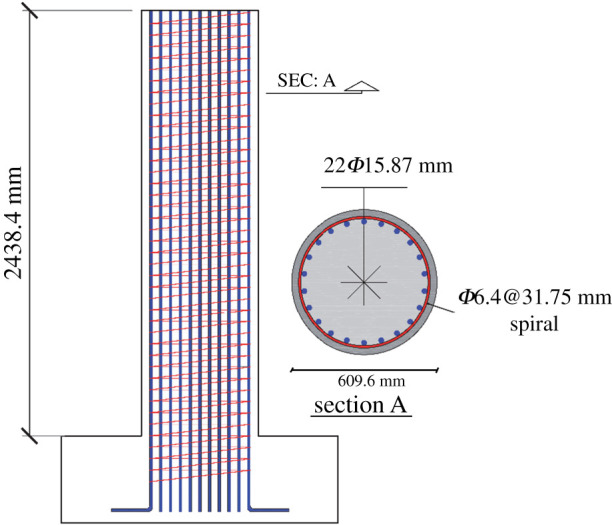
Structural details of the case-study RC column. (Online version in colour.)

In this study, a benchmark RC column is selected from the UW–PEER experimental test database [59]. The structural details of this column are shown in figure 5. As figure 5 shows, the proposed column is a cantilever flexural-governed circular RC column with an aspect ratio of 4. This column is 2438.4 mm in height and 609.6 mm in diameter; the section is reinforced with 15.87 mm diameter bars that are symmetrically distributed along the perimeter of the section (22 bars in total) and confined with a 6.4 mm spiral with a maximum spiral pitch of 31.75 mm. The yield stress and maximum stress of steel reinforcements are 497 MPa and 662 MPa, respectively; the fracture strain of spirals and longitudinal reinforcement are 0.16 and 0.195, respectively. The cylindrical compressive strength of concrete is 31 MPa, and the axial force on top of the column is 653.8 kN.

### (b) Fibre-based finite-element model

**Figure 6.**
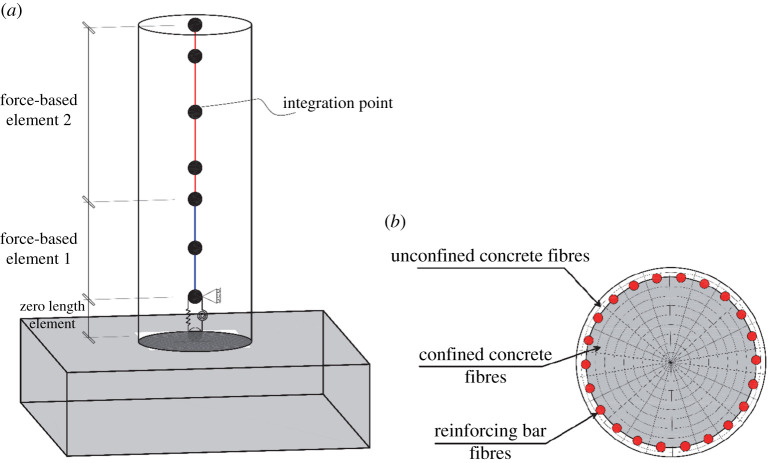
Finite-element model of the case-study RC column: (*a*) force-based elements and (*b*) fibre section. (Online version in colour.)

To simulate the nonlinear structural behaviour of the case-study RC bridge pier, the open system for earthquake engineering simulation (OpenSees) is employed [60]. It provides a platform to create nonlinear finite-element models for simulating the response of structural systems. Figure 6*a* shows the proposed finite-element model of the case-study RC column. As figure 6*a* shows, the whole length of the column is divided into two force-based elements. Force-based element 1 includes three integration points and force-based element 2 includes five integration points with the Gauss–Lobatto integration scheme. At each integration point, a fibre section is assigned to the model, where the cross-section of the column is discretized into several unconfined concrete, confined concrete and reinforcing bars fibres (figure 6*b*). To address the strain localization issue [61,62], the length of force-based element 1 is adjusted such that the integration length of the bottommost integration point (which is the most critical section of the column) becomes equal to the effective buckling length of longitudinal reinforcement. Detailed information about the calculation of the buckling length is available in [63]. To capture the strain penetration effects and slippage of reinforcement at the adjacent of connection of the column to the footing, a zero-length section element is used [64]. This modelling technique has been validated successfully against experimental results [63].

The accuracy of numerical models in the simulation of nonlinear structural behaviour significantly depends on the accuracy of nonlinear material models. In this study, the nonlinear stress–strain behaviour of reinforcing steel bars is simulated using the phenomenological buckling model developed by Kashani *et al*. [65]. This model simulates the inelastic buckling and cumulative fatigue degradation of reinforcing bars. More details on this model are available in [65,66]. To model the nonlinear response of concrete cover, the Popovics concrete material (uniaxial *Concrete04* material model) [67] available in OpenSees is employed. Based on the study of Karsan–Jirsa [68], this model simulates linear unloading/loading stiffness degradation and exponential decay of tensile strength. To consider the positive influence of confinement on the compressive strength and ductility of core confined concrete, the model proposed by Mander *et al*. [69] is considered. In §3e(i) of this paper, the proposed modelling technique and structural details will be used to carry out the nonlinear static analyses.

#### (i) Modelling the impact of corrosion on reinforcing steel

Chloride-induced corrosion of steel reinforcing bars is initiated once the protective thin oxide film around the bars is de-passivated due to the excessive concentration of chloride ions [70]. In the literature, a wide range of experimental studies confirmed that corrosion reduces both the strength and ductility of steel reinforcement. Du *et al*. [71,72] proposed the following empirical linear equation to modify the mechanical properties of corrosion-damaged reinforcement

3.1 $$Q_{\text{corr}}=[1-\eta \psi (w)]Q_{\text{uncorr}},$$

where *Q*_corr_ and *Q*_uncorr_ represent the mechanical properties of corroded and uncorroded reinforcement, respectively, and *η* is the pitting coefficient. The value of *η* for yield stress, ultimate stress and rupture strain of reinforcement is 0.005, 0.005 and 0.035, respectively. Therefore, having *ψ*(*w*) for each *w*, the tensile mechanical properties of steel reinforcement can be updated with time.

**Figure 7.**
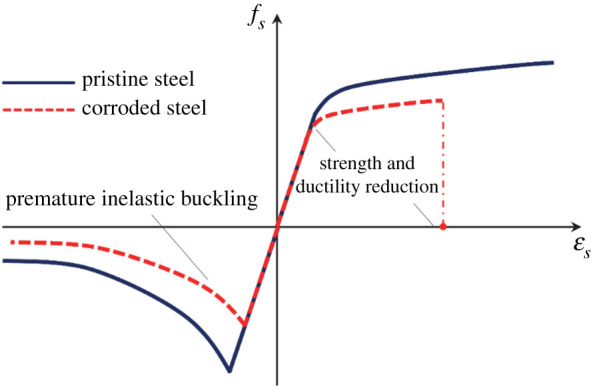
Stress–strain model of corroded and pristine reinforcing bars. (Online version in colour.)

Previous studies [73–76] show that, besides the tensile mechanical properties, corrosion causes premature inelastic buckling and low-cycle fatigue failure of reinforcing bars. To capture this phenomenon in numerical modelling, Kashani *et al*. [65] developed a buckling model, where the compressive yield stress of corroded reinforcement is modified in terms of *ψ*(*w*) and slenderness ratio of reinforcement. Further details are available in [65]. Figure 6 shows an overview of the adopted backbone stress–strain model of reinforcing bars in tension and compression. As figure 7 shows, the considered model of reinforcing bars accounts for the corrosion-induced strength and ductility reduction (in tension) as well as premature inelastic buckling (in compression).

#### (ii) Impact of corrosion on cracked concrete cover and core confined concrete

**Figure 8.**
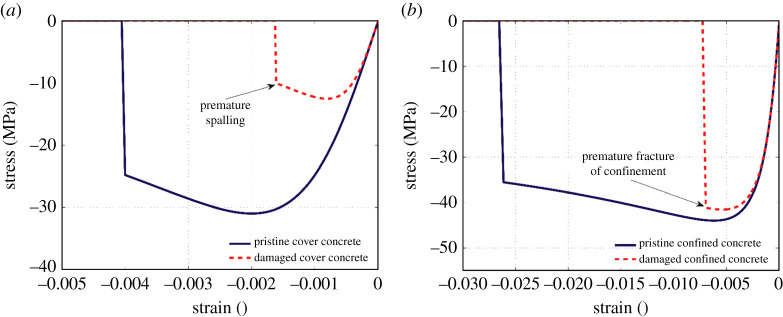
Stress–strain model of concrete: (*a*) unconfined concrete and (*b*) confined concrete. (Online version in colour.)

The expansive corrosion products around the corroded reinforcement cause internal tensile stress in the concrete cover. Once this tensile stress exceeds the tensile strength of the cover concrete, several cracks are formed along the length of reinforcing bars [77]. Development of these corrosion-induced cracks finally results in premature spalling of cover concrete. In numerical modelling, to account for this phenomena, the compressive strength and associated strain with spalling of concrete cover are modified based on the methodology proposed by Coronelli & Gambarova [78]. Details of this methodology are available in [78]. Figure 8*a* schematically compares the simulated stress–strain response of corrosion-damaged concrete cover with that of pristine concrete cover.

In the absence of a reliable methodology in the literature, in this study, the nonlinear stress–strain response of corrosion-damaged confined concrete is modified using a simplified procedure. This method suggests that the mechanical properties and volumetric ratio of the transverse reinforcement should be modified to account for the negative influence of corrosion. In this way, the ultimate compressive strain and compressive strength of corrosion-damaged confined concrete will be reduced. Further details are available in [79,80].

Figure 8*b* shows an example of the nonlinear response of corrosion-damaged confined concrete versus the corresponding response of pristine confined concrete.

### (c) Random variables and Monte Carlo simulation

A probabilistic approach is employed to reflect the inherent uncertainty in the deterioration process. In this study, the parameters involved with equations (2.8–2.18) for the estimation of *t*_cr_, *t_p_*(*w*) and *ψ*(*w*), as well as mechanical and geometrical properties of the case-study structure are considered as random variables. Table 2 summarizes the statistics of the considered random variables. For each random variable, 300 random samples are generated using Latin hypercube sampling (LHS) method [86]. These random variables will be used in §3d to construct the proposed Markov chain model and in §3e to conduct SVA. It should be noted that future work might consider other sources of uncertainty beyond the scope of this case study. For example, accounting for error in a given predictive model and/or epistemic uncertainties in the choiceof model.

**Table 2.** Statistics of random variables or other deterministic input parameters used in the deterioration model and finite-element modelling.

parameter	symbol	distribution	mean value	unit	COV	reference
compressive strength of concrete	*f_c_*	lognormal	31	MPa	0.16	[81]
yield strength of longitudinal reinforcement	*f_y_*	lognormal	497	MPa	0.05	[82]
yield strength of spirals	*f_ys_*	lognormal	606.8	MPa	0.05	[82]
concrete cover to surface of longitudinal reinforcements	*X*	normal	25	mm	0.205	[46]
diameter of longitudinal reinforcement	*d_l_*	normal	15.9	mm	0.02	[83]
diameter of spirals	*d_s_*	normal	6.4	mm	0.02	[83]
rupture strain of reinforcement	*ε_r_*	normal	0.19	—	0.09	[84]
thickness of the porous zone	*t*_pore_	lognormal	12.5	μm	0.2	[52]
density of corrosion product	*ρ*_rust_	normal	3600	kg m^−3^	0.1	[52]
density of steel	*ρ*_steel_	normal	7850	kg m^−3^	0.1	[52]
molecular weight of steel to that of corrosion products	*α*	deterministic	0.57	—	—	[46]
creep coefficient of the concrete	*φ*	deterministic	2	—	—	[46]
Poisson's ratio of the concrete	*ν*	deterministic	0.2	—	—	[46]
water to cement ratio	*W/C*	deterministic	0.5	—	—	[85]

### (d) Estimation of the condition state probabilities

After the generation of random variables, in this section, the time-variant probability of being in each predefined CS (defined in §2b) is calculated using the predictive Markovian process described in §2a. Using equation (2.15), 300 random duration times (*T*) are obtained for each CS by each set of random variables from table 2. Using the crack width leaving threshold of each CS defined in §2b (e.g. *w* = 1 mm for CS_3_), the cumulative distribution function of *T_i_* (i.e. *F_i_*(t)) is simulated per CS. It should be noted that for CS_1_, where the *w* = 0, the time for crack initiation (*t*_cr_) is used as the threshold of leaving this state, and therefore, equation (2.15) is used to produce random times from corrosion initiation to crack initiation (i.e. *t*_cr_* *= *T*_1_).

**Figure 9.**
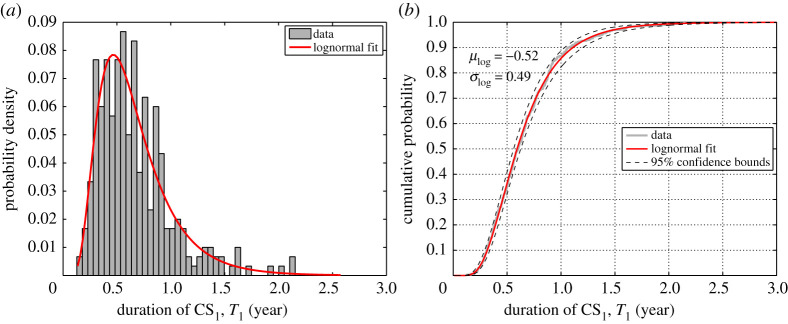
Comparison of simulated data and fitted lognormal distribution for duration of CS_1_, *T*_1_: (*a*) histogram and (*b*) cumulative distribution. (Online version in colour.)

As an example, figure 9 shows the histogram and the simulated cumulative probability (*F_1_*(*t*)) of the generated random CS_1_ durations (*T*_1_)*.* Among different tested distributions, the lognormal distribution function is found to be the best fit to the simulation data for duration of each CS (*T_i_*). The goodness of fit is evaluated by employing the Kolmogorov–Smirnov (K–S) test [87]. It is found that all the random *T_i_* variables (CS durations) pass the K–S test at the significance level of 0.05 when comparing the empirical (simulated) versus theoretical distribution function. Furthermore, the 95% confidence intervals are shown in figure 9 relative to the simulated data, where most all simulated data fall within the confidence interval. Therefore, the lognormal distribution is fitted to the estimated values of *T_i_*. This is in a good agreement with the outcome of an investigation carried out by Andrade *et al*. [88] on the naturally corroded beam and column specimens. The model parameters of fitted lognormal distributions for duration of each CS are summarized in table 3. In table 3, *μ*_log_ and *σ*_log_ are the mean and standard deviation of the fitted lognormal distribution function.

**Table 3.** Statistics of lognormal distribution for duration of each CS.

model parameter	*T*_1_ (year)	*T*_2_ (year)	*T*_3_ (year)	*T*_4_ (year)	*T*_5_ (year)
*μ*_log_	−0.52	1.33	1.95	2.62	3.03
*σ*_log_	0.49	0.25	0.23	0.23	0.22

The fitted lognormal cumulative distribution functions of each CS are used to calculate *R_i_*(*t_p_*, Δ) (equation (2.4)). Therefore, having *R_i_*(*t_p_*,Δ) at each *t_p_*, the time-dependent Markov transition probability matrix (equation (2.5)) can be calculated at each time step; then, from equation (2.6), the time-dependent state probabilities can be obtained.

In table 4, as an example, the calculated Markov transition probabilities for the last month of *t_p_* = 5 years are provided. The time increment Δ = 1 month is used in the calculations.

**Table 4.** Markov transition probability matrix for the last month of *t_p_* = 5 years.

condition state	CS_1_	CS_2_	CS_3_	CS_4_	CS_5_
CS_1_	0.8568	0.1432	0	0	0
CS_2_	0	0.8956	0.1044	0	0
CS_3_	0	0	0.9887	0.0113	0
CS_4_	0	0	0	1	0
CS_5_	0	0	0	0	1

**Figure 10.**
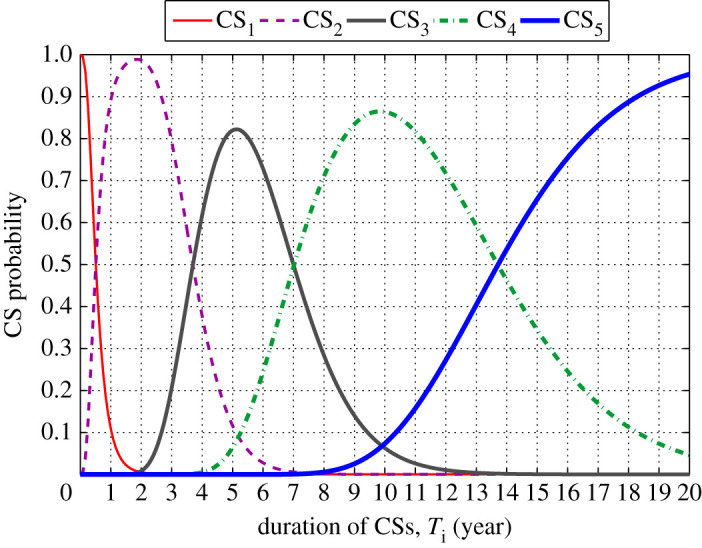
Variation of CS probabilities as a function of time. (Online version in colour.)

Figure 10 shows the variation of the state probability for each considered CS. It can be seen from this figure that while the probability of remaining in a CS generally gets lower as time increases, the probability of reaching a more severe CS increases with time. For example, while the probability of being in CS_1_ at the onset of corrosion initiation is nearly 100%, it drops to around 10% after 1 year from corrosion initiation. At the same time, the structure is more likely to be in the next state (i.e. CS_2_). Moreover, it can be seen from figure 10 that the structure is less likely to be in CS_5_ up to 8 years from corrosion initiation. However, beyond this time, the probability of being in this CS gradually increases over time.

In §3e(iii), the time-variant state probabilities shown in figure 10 will be used in conjunction with the SVA to establish a relationship between visual condition damage state to residual structural performance.

### (e) Structural vulnerability analysis, results and discussion

#### (i) Nonlinear pushover analysis

**Figure 11.**
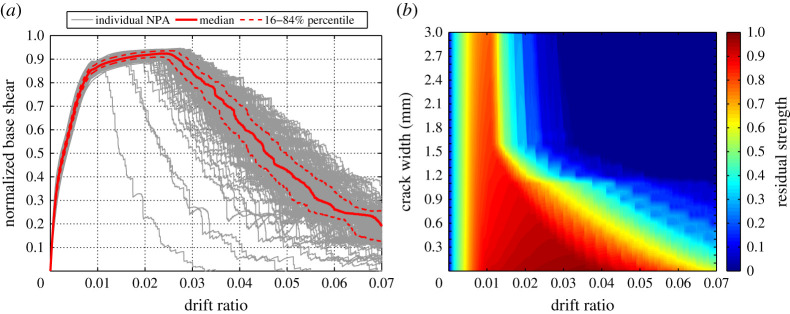
Example NPA results for: (*a*) all the established FE models for *w* = 0.5 mm and (*b*) an FE model (one combination out of 300 different combinations). (Online version in colour.)

In this section, using the structural details of the case-study RC bridge pier and the modelling technique described in §3b of this paper, nonlinear pushover analyses are performed to evaluate the time-dependent performance of the structure as corrosion-induced cracking is progressively increased. To this end, a wide range of corrosion-induced cracks from 0 to 3 mm with the steps of 0.1 mm is considered (31 crack sizes in total). For each crack size, using equation (2.18) and each combination of random variables defined in table 2, 300 random mass losses as a function of crack width (*ψ*(*w*)) are generated. For each *ψ*(*w*), the material properties of the proposed structure are modified, and hence, for each crack size, 300 statistically different but nominally identical FE models are established. This results in a total of 9300 NPAs. To perform the analyses, the base of the column is considered to be fully fixed and the tip of the column is free to be moved in each direction. Moreover, the second-order effect due to the axial load and large lateral displacement is included in the analyses by employing P-delta coordinate transformation object available in the OpenSees. During the analyses, the tip displacement and base shear of the column, as well as the material response of concrete and steel bars, are recorded. Figure 11*a* shows, as an example, the NPA results of all the 300 established FE models for *w* = 0.5 mm. In this figure, the base shear output of each model is divided by its corresponding base shear of the uncorroded model (at *w* = 0) to obtain the normalized base shear. Moreover, the recorded tip displacements of the column are divided by the column height to obtain the drift ratio. To show the variability of the results, the median and 16–84% percentiles curves are shown on the top of individual NPA results. Figure 11*a* shows that while there is not a significant variability at the initial loading steps (up to 0.01 drift ratio), results are highly variable in the post-peak region. As another example, figure 11*b* shows the NPA results of FE models established using one combination (out of 300 different combinations) of generated random variables for all the considered range of crack sizes (i.e. 31 NPAs in total). In this figure, the variation of residual strength (*S*_corr_/*S*_uncorr_) is plotted against the drift ratio of the column and different corrosion-induced crack sizes. Figure 11*b* indicates that both the residual strength and the tolerable drift ratio (and as a result residual ductility) of the column significantly reduces as the crack size increases.

Using the outputs of NPAs, the displacement ductility of an RC structure (*μ*) can be calculated as a ratio of the ultimate displacement (Δ*_u_*) to the yield displacement Δ*_y_*, where Δ*_u_* corresponds to the crushing of confined concrete or fracture of longitudinal bars (whichever takes place first) and Δ*_y_* corresponds to the yielding of longitudinal reinforcement. Therefore, *μ*_loss_ can be calculated as 1 − *μ*_corr_/*μ*_uncorr_, where *μ*_corr_ is the ductility of the corroded structure (beyond the *t_p_* = 0); and *μ*_uncorr_ is the corresponding ductility of the uncorroded structure (at *t_p_* = 0). Similarly, *S*_loss_ is calculated as 1 − *S*_corr_/*S*_uncorr_, where *S*_corr_ is the maximum base shear of the corroded structure, and *S*_uncorr_ is the maximum base shear of the corresponding uncorroded structure.

In this study, four representative ductility performance limit states (DPLs) as defined in table 5 and three strength performance limit states (SPLs) as defined in table 6 are considered. It should be noted that the structure is considered to fail beyond 60% ductility loss or 20% strength loss [89].

**Table 5.** Ductility loss performance limit states.

performance limit	symbol	performance limit state
performance limit 1	DPL_1_	0 < *μ*_loss_ ≤ 15%
performance limit 2	DPL_2_	15% < *μ*_loss_ ≤ 25%
performance limit 3	DPL_3_	25% < *μ*_loss_ ≤ 35%
performance limit 4	DPL_4_	35% < *μ*_loss_ ≤ 45%
performance limit 5	DPL_5_	45% < *μ*_loss_ ≤ 60%

**Table 6.** Strength loss performance limits states.

performance limit	symbol	performance limit state
performance limit 1	SPL_1_	0 < *S*_loss_ ≤ 5%
performance limit 2	SPL_2_	5% < *S*_loss_ ≤ 10%
performance limit 3	SPL_3_	10% < *S*_loss_* ≤ *15%
performance limit 4	SPL_4_	15% < *S*_loss_ ≤ 20%

**Figure 12.**
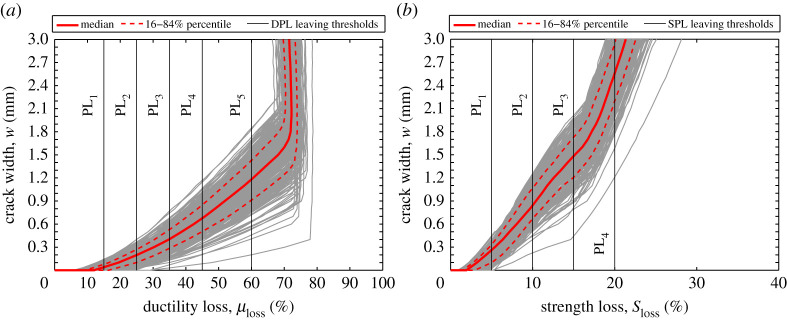
Ductility loss (*a*) and strength loss (*b*) versus corrosion-induced crack width. (Online version in colour.)

From the outputs of each NPA (out of 9300 analysis), *μ*_loss_ and *S*_loss_ are calculated. Figure 12*a* shows the calculated ductility loss percentages versus corrosion-induced crack size. In this figure, each curve represents the variation of ductility loss of each random variable combination corresponding to different crack sizes. As figure 12*a* shows, the variability of ductility loss for a given crack width is very high, as a reflection of the significant uncertainty associated with the stochastic deterioration process. For example, for *w* = 1 mm, the ductility loss varies from approximately 40 to 78%. To summarize the results, in figure 12*a*, the median and 16–84% percentile curves of the ductility loss percentages are plotted. The figure shows that at the onset of initiation of corrosion-induced cracks, the ductility of the structure has already been reduced by 10%. After the formation of cracks, the ductility loss increases up to around 70% as crack width increases. However, beyond approximately 1.7 mm median crack width, the ductility loss remains approximately constant for larger cracks. The leaving thresholds of each DPL are mapped to figure 12*a* by vertical solid lines. These thresholds are used in the next section to perform SVA. Figure 12*b* shows the strength loss of the case-study structure associated with the various corrosion-induced cracks. Figure 12*b* shows that for the considered set of corrosion-induced cracks, the median of strength loss varies from 0 to approximately 22%. Similarly, this figure shows that the variability of strength loss for a given crack width is significantly high. For example, for *w*=1 mm, it varies from 8 to 18%. The leaving thresholds of the predefined SPLs shown in this figure are used in the next section to carry out the SVA.

#### (ii) Structural vulnerability analysis

Using the proposed SVA methodology described in §2d and the outputs of the NPAs, the probability of exceeding each performance limit state is quantified in this section as a function of *w.* Here, the number of random samples is 300 (*n* = 300). The K–S goodness-of-fit test, similar to what was shown in figure 9 for CS duration times, confirms the validity of the lognormal distribution at the significance level of 0.05.

**Figure 13.**
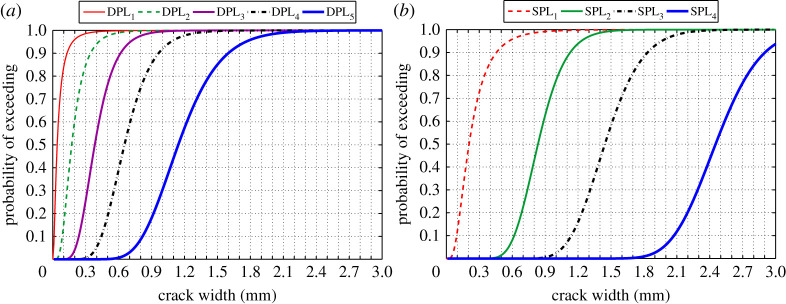
Probability of exceeding: (*a*) DPLs and (*b*) SPLs. (Online version in colour.)

Figure 13*a,b* shows the probability of exceeding each DPL and SPL, respectively, against corrosion-induced crack width. It can be seen from figure 13*a* that the probability of exceeding the predefined ductility loss limit states increase sharply as crack width increases. As discussed earlier in §2*b*, *w* = 0.5 mm and *w* = 1 mm have been proposed in the literature as the serviceability failure limit state. Figure 13*a* shows that, for example, at *w* = 0.5 mm the probability of exceeding 35% ductility loss (DPL_3_) is approximately 75%, and figure 13*b* shows that the probability of exceeding 10% strength loss (SPL_2_) is about 2%. For *w* = 1 mm, the corresponding probabilities are 100% and 78%, respectively. These simple comparisons show that the corrosion-induced cracking reduces the ductility of corroded RC structures much more than their strength. Moreover, as figure 12 shows, at the onset of *w* = 0.5 mm, the variation of ductility and strength losses are not significant; therefore, from the structural engineering point of view, *w* = 0.5 mm can be a good conservative estimation of serviceability limit state.

The results presented in this section are used in the next section to explore the relationship between the SVAs and Markov chain CS probabilities.

#### (iii) Relationship between structural vulnerability analysis results and condition state probabilities

**Figure 14.**
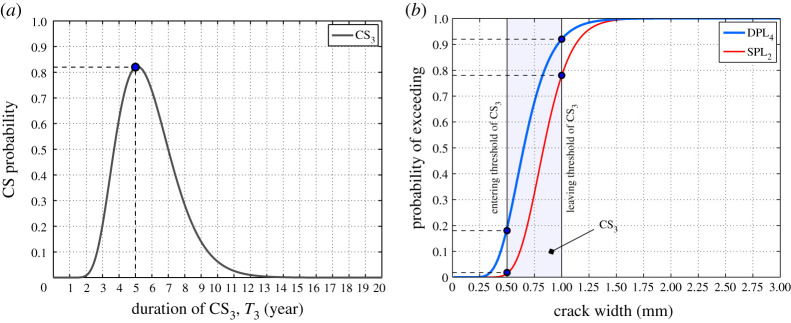
An example of linking structural performance to the Markovian representation of deterioration. (Online version in colour.)

To quantitatively relate the performance of the structure to the Markovian representation of deterioration (in terms of corrosion-induced crack size), one effective approach is to relate the CS probabilities (figure 10) to the probability of exceeding predefined structural PLs (figure 13*a*,*b*). For example, figure 14*a* shows that after *t_p_* = 5 years, the probability of being in CS_3_ (where 0.5  ≤ *w* ≤ 1 mm) is 80%. On the other hand, as figure 14*b* shows at 0.5 <* w *≤ 1 mm, the probability of exceeding ductility loss of 45% (DPL_4_) is between 18% and 92%, respectively. At the same time, the probability of exceeding 10% strength loss (SPL_2_) is approximately 2% and 78%, respectively (figure 14*b*). In this way, the width of measured corrosion-induced cracks on site can be directly related to the structural performance in terms of ductility or strength. This is very important for bridge owner and manager to prioritize critical bridges within a bridge network for an optimize and safe maintenance scheme. It should be noted that the comparative results given here are only an example, and this methodology can be applied to other structural components or the whole bridge structure. The current study provides a Markovian deterioration process to link the observable deterioration level (measured inspection data) and structural performance. Furthermore, in the future research, the proposed model can be used in combination of inspection data and/or structural health monitoring data (e.g. [90]) for further model validation and calibration.

## 4. Conclusion

In this paper, a Markovian deterioration model is proposed to predict the future CS of corroded RC bridges. To this end, a discrete-time Markov chain model is presented to predict the CS of corroded bridges based on the corrosion-induced crack width, which can be measured on site during the inspection. Using SVA, the time-dependent structural performance of deteriorated structures is related to the corrosion-induced crack-based CS probabilities. Finally, the application of the proposed methodology was demonstrated through a case-study corroded RC bridge pier. It was indicated that SVA can be effectively linked to the Markovian representation of observable corrosion damage to predict the probability of exceeding a specific performance level at a given time. This was illustrated using an example, where, the time-dependent probability of the structure to be in a CS was estimated using the proposed Markov chain process. Subsequently, the probability of exceeding predefined performance limit states corresponding to upper and lower bounds of the same CS was estimated using SVA.

The proposed methodology could be an effective tool to improve the concept of bridge condition index which is currently calibrated for a specific bridge using the inspection outcome of expert engineers. The improved bridge condition index can help decision-makers to address the maintenance funding prioritization within an entire bridge stock. It should be noted that the main focus of the current study was at the component level. Using extensive inspection data from a broad inventory of existing deteriorated bridges, the future study can be concentrated on the extension of the current methodology from component level to the system level. This can provide a platform to anticipate, for example, how the given extent of damage in a component can affect the failure probability of a system at a given time in the future. Nevertheless, this study provides a foundation for future research in time-dependent predictive SVA of corroded bridges using Markov chain model.

## Data accessibility

The OpenSees and MATLAB codes are available in the electronic supplementary material.
